# Plasmacytoid Dendritic Cells Contribute to the Production of IFN-β via TLR7-MyD88-Dependent Pathway and CTL Priming during Respiratory Syncytial Virus Infection

**DOI:** 10.3390/v11080730

**Published:** 2019-08-08

**Authors:** Tae Hoon Kim, Dong Sun Oh, Hi Eun Jung, Jun Chang, Heung Kyu Lee

**Affiliations:** 1 Graduate School of Medical Science and Engineering, Korea Advanced Institute of Science and Technology (KAIST), Daejeon 34141, Korea; 2 Department of Internal Medicine, Gyeongsang National University School of Medicine and Gyeongsang National University Changwon Hospital, Changwon 51472, Korea; 3 Graduate School of Pharmaceutical Sciences, Ewha Womans University, Seoul 03760, Korea; 4 KAIST Institute for Health Science and Technology, KAIST, Daejeon 34141, Korea

**Keywords:** RSV, plasmacytoid dendritic cell, dendritic cell, type I IFN, cytotoxic CD8^+^ T cells

## Abstract

Respiratory syncytial virus (RSV) is the leading cause of respiratory viral infection in infants and children, yet little is known about the antiviral response of plasmacytoid dendritic cells (pDCs) to RSV infection. We tracked the cellular source of interferon-β using interferon-β/yellow fluorescent protein (YFP) reporter mice and identified the signaling pathway activated by RSV that induces type I interferon production in pDCs and DCs. Results from in vitro analyses of RSV-stimulated bone marrow cells revealed that RSV induces interferon-β production in both pDCs and DCs. Kinetic analyses of interferon-β-producing cells in RSV-infected lung cells in vivo indicated that pDCs are rapidly recruited to sites of inflammation during infection. These cells produced interferon-β via the TLR7-MyD88-mediated pathway and IFNα1R-mediated pathway rather than the MAVS-mediated pathway. Moreover, pDC-ablated mice exhibited decreased interferon-γ production and the antigen specificity of CD8^+^ T cells. Collectively, these data indicate that pDCs play pivotal roles in cytotoxic T lymphocyte (CTL) responses and are one of producers of type I interferon during RSV infection.

## 1. Introduction

Respiratory syncytial virus (RSV) is a major cause of respiratory illness in young children, causing recurrent childhood wheezing or asthma [1,2]. Although most infants are infected with RSV at least once within the first 2 years of life, many suffer repeated infections throughout life, as well as an increased risk of morbidity and mortality. RSV is also an important cause of opportunistic respiratory infections in elderly persons, immunocompromised patients, and cardiopulmonary patients.

Production of type I interferon is an immediate innate immune response to viral infection and is essential for the antiviral response; it modulates the expression of various genes involved in host defense and initiates the adaptive immune response. Plasmacytoid dendritic cells (pDCs) have been known to express higher levels of type I interferon than other immune cells following viral stimulation, and several reports have shown that RSV can induce type I interferon production [3,4,5,6]. Nonetheless, the in vivo role of pDCs in RSV infection remains unclear.

Viral infection or uptake of virus by epithelial cells, pDCs, and dendritic cells (DCs) precipitates a direct antiviral response via the production and release of cytokines and chemokines, initiating an adaptive immune response. These cells have pattern-recognition receptors (PRRs), toll-like receptors (TLRs), retinoic acid-inducible gene-I (RIG-I)-like receptors, and nucleotide-binding oligomerization domain-like receptors that recognize pathogen-associated molecular patterns on invading pathogens [7,8]. These immune cells have different levels of PRRs, and the cell type-specific expression of PRRs plays unique roles in antiviral immunity. Previously, we presented MyD88, but not TLR7, as important molecules for RSV recognition and type I IFN and pro-inflammatory cytokine production in DCs and macrophages [9]. However, the specific functions of these PRRs in pDCs on RSV infection are poorly defined.

Here, we show that pDCs and DCs, are sources of interferon-β in RSV infection of interferon-β/YFP reporter mice. We also investigate the signaling pathway activated by RSV to produce type I interferon and examine the roles of pDCs in adaptive T cell immunity against RSV infection.

## 2. Materials and Methods

### 2.1. Animals

Interferon-β/YFP reporter (B6.129-Ifnb1tm1Lky/J) [10], MyD88^−/−^ (B6.129P2(SJL)-Myd88tm1.1Defr/J) [11], interferon-αR1^−/−^ (B6(Cg)-Ifnar1tm1.2Ees/J) [12], TLR7^−/−^ (B6.129S1-Tlr7tm1Flv/J) [13], MAVS^−/−^ (B6;129-Mavstm1Zjc/J) [14], and BDCA2-DTR (C57BL/6-Tg(CLEC4C-HBEGF)956Cln/J) [15] mice were obtained as previously described. TLR7^−/−^, MAVS^−/−^, and BDCA2-DTR mice were purchased form Jackson Laboratory (Maine). All mice were housed in a specific pathogen-free facility at KAIST. The study protocol was approved by the Institutional Animal Care and Use Committee (IACUC) of Korea Advanced Institute of Science and Technology (KAIST). This study was approved by the IACUC of KAIST (KA2013-55). Gender- and age-matched mice between 8 and 12 weeks of age were used for the experiments.

### 2.2. RSV Infection and pDC Depletion In Vivo

The A2 RSV strain was grown in HEp-2 cells and titrated for infectivity as described previously [16,17]. Mice were anaesthetized by intraperitoneal injections of 80 mg/kg ketamine (Youhanyanghaeng) and 16 mg/kg xylazine (BAYER Korea) before intranasal inoculation with 1.0 × 10^7^ plaque-forming units (pfu) of RSV. To ablate pDCs, WT and BDCA2-DTR mice were intraperitoneally treated with 250 ng/mouse of diphtheria toxin (DT) (Sigma Aldrich, St. Louis, MO, USA) 1 day prior to infection with RSV, and 1 and 3 days post infection to maintain pDC depletion.

### 2.3. RSV Infection In Vitro and Cytokine Measurements

To obtain bone marrow (BM) cells in mice, femurs and tibiae were removed from each mice and BM were plunged by using 10 mL syringe with 10 mL of serum-free DMEM and passed through in to 70 μm cell strainer. Next, BM cells were counted with hemo-cytometer after erythrocytes lysis with in-house made ACK lysis buffer (150 mM NH_4_Cl, 10 mM KHCO_3_, 0.1 mM Na_2_EDTA in 3’DW). 2 × 10^5^ BM cells were stimulated with live or inactivated RSV or 2.5 μg of CpG_2216_ (Invitrogen, Carlsbad, CA, USA) in 200 μL of 10% FBS containing RPMI 1640 media in 96 well plat bottom cell culture plate (Corning) for 18 h. Cell-free supernatants were collected, and IL-6 (BD Biosciences, San Jose, CA, USA ), IL-12p40 (eBioscience, San Diego, CA, USA), and interferon-β (BioLegend, San Diego, CA, USA) levels were analyzed using specific ELISA kits, according to the manufacturer’s instructions.

### 2.4. Preparation of Single Lung Cell Suspensions

To obtain single lung cell suspensions, isolated lung samples were prepared according to published procedures [18]. Briefly, lungs were removed from each mice, minced into small pieces, and then digested with 2 mg/mL collagenase IV (Worthington Biochemical Corp, Lakewood, NJ, USA) and 30 µg/mL DNase I (Roche, Basel, Switzerland) in DMEM for 30 min at 37 °C. Digested lung pieces were then passed through 70-µm cell strainers prior to Percoll (GE Healthcare, Marlborough, MA, USA) density-gradient (30/70%) centrifugation to isolate the leukocytes and remove debris. Erythrocytes were lysed using an in-house ACK lysis buffer. The resulting cells were subjected to flow cytometry.

### 2.5. Flow Cytometry

Single-cell suspensions were first pretreated with anti-CD16/32 (2.4G2) antibodies to block Fc receptors and then stained with anti-CD11c (HL3), anti-MHC class II (M5/114.15.2), anti-CD317 (927), anti-Siglec-H (551), or anti-CD45.2 (104) antibodies. Live cells were gated based on 4’,6-diamidino-2-phenylindole (DAPI) (Invitrogen, Carlsbad, CA, USA) exclusion. Multiple cell populations were separated by flow cytometry (LSR Fortessa or Calibur; BD Biosciences). H-2D_b_ tetramers specific for the RSV M_187–195_ peptide (NAITNAKII) were prepared with streptavidin-allophycocyanin (APC), using the protocol of the NIH Tetramer Core Facility (https://tetramer.yerkes.emory.edu/support/protocols) [17]. Single-cell suspensions from the lungs of immunized mice were pretreated with anti-CD16/32 (2.4G2) antibodies to block Fc receptors and then stained with anti-CD8α (53-6.7) and anti-CD3e (145-2C11) antibodies. Then, APC-labelled tetramer staining was performed. The number of RSV M_187–195_ peptide-specific CD8^+^ T cells was analyzed by flow cytometry (Calibur; BD Bioscience). Final analysis and graphical output of the results were obtained using FlowJo software (Version 9, Tree Star, Inc., Ashland, OR, USA).

### 2.6. CD4^+^ and CD8^+^ T Cell Responses

RSV-specific T cell responses were analyzed. At 8 days post-infection, CD4^+^ and CD8^+^ T cells were isolated from the spleens of infected mice using anti-CD4 and anti-CD8 microbeads (Miltenyi Biotec, Bergisch Gladbach, Germany), respectively, according to the manufacturer’s instructions. 2 × 10^5^ of CD4^+^ and CD8^+^ T cells were then stimulated with the indicated amounts of heat-inactivated RSV virions or RSV M_187–195_ peptides and cocultured with 2 × 10^5^ wild type splenocytes for 72 h at 37 °C. Interferon-γ production in the supernatants was measured by ELISA (eBioscience).

### 2.7. RSV Titration

RSV titer in lung was assessed by plaque assay [9,17]. Briefly, RSV-infected mice were sacrificed by carbon dioxide gas and lungs were harvested and stored in PBS. Lungs were homogenized by passing the spleen through a 70-μm cell strainer, and RSV titers in harvested lung tissue homogenate were determined using plaque assay on Hep-2 cell monolayers.

### 2.8. Statistics

The data are presented as the mean ± standard error of the mean. Statistical significance was evaluated with two-tailed, unpaired Student’s *t* tests using Prism software (GraphPad 7.0). *P* values less than 0.05 were considered statistically significant.

## 3. Results

### 3.1. pDCs Produce Higher Levels of Interferon-β than DCs during RSV Infection In Vitro

**Figure 1:**
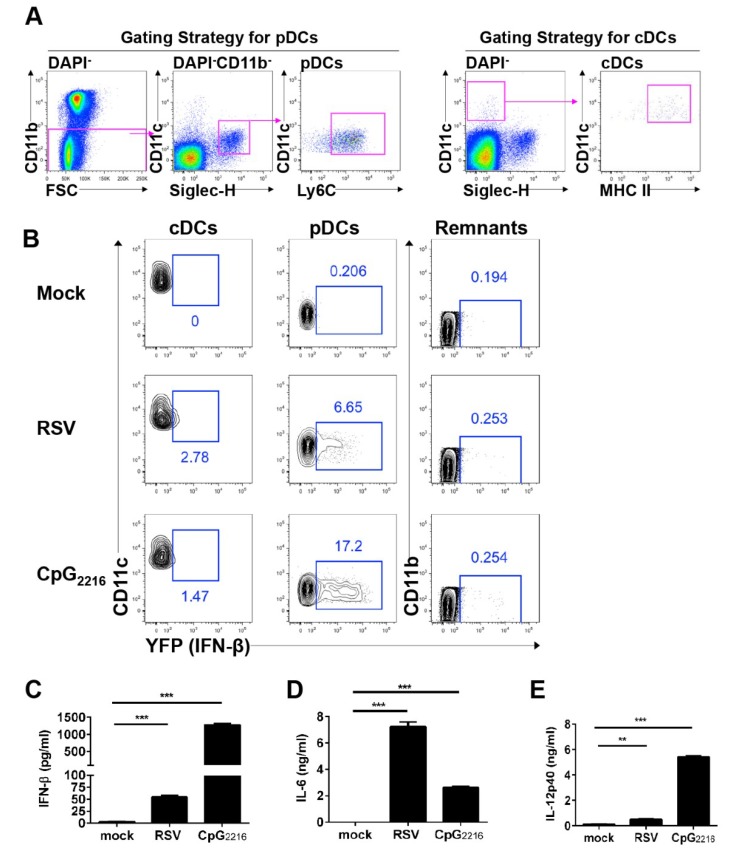
cDCs and pDCs in BM can produce interferon-β during RSV infection. (**A**) Flow cytometric gating strategy for cells. Representative dot plots showing cDCs in the CD11c^hi^Siglec-H^−^MHC-II^hi^ and pDCs in the CD11b^lo^CD11c^int^Siglec-H^+^Ly6C^+^ populations, gated on CD45.2^+^DAPI^−^. (**B**) BM cells from interferon-β/YFP reporter mice were infected with RSV at a multiplicity of infection of 3 or with 2.5 μg/mL CpG2216. Cells were harvested 18 h after stimulation and analyzed for the expression of interferon-β by flow cytometry. The remaining cells (remnants) were defined as the population of CD11b^−^CD11c^−^Ly6C^−^ cells gated on DAPI^−^. (**C**–**E**) BM cells were infected with RSV at a multiplicity of infection value of 3 or with 2.5 μg/mL CpG2216. The supernatant was collected 18 h after stimulation and analyzed for interferon-β (**C**), IL-6 (**D**), and IL-12p40 (**E**) by ELISA. The data are presented as the mean ± SEM and representative of three independent experiments. * *P* < 0.05, ** *P* < 0.01, and *** *P* < 0.001 as calculated by the Student’s t test.

It is known that both pDCs and DCs can produce type I interferon during RSV infection [19,20]. To identify the cell types that produce type I interferon in vitro, we used the interferon-β/YFP reporter mouse, a reliable tool for the visualization and spatiotemporal tracking of interferon-β-producing cells. We infected BM cells with RSV and measured interferon-β production in each cell population (Figure 1A). Our results showed that pDCs produced interferon-β in response to RSV infection (Figure 1B). A small proportion of DCs also produced interferon-β (Figure 1B). No other cells produced interferon-β in response to RSV infection. To determine whether YFP^+^ cells were capable of type I interferon secretion, we measured interferon-β and proinflammatory cytokine levels in culture supernatants of RSV-infected BM cells. As expected from our flow cytometric analysis, RSV-infected BM cells secreted interferon-β and proinflammatory cytokines (Figure 1C–E). Taken together, pDCs are the predominant interferon-β producing cell type during RSV infection in vitro.

### 3.2. pDCs and cDCs are the Interferon-β Producers during RSV Infection In Vivo

**Figure 2:**
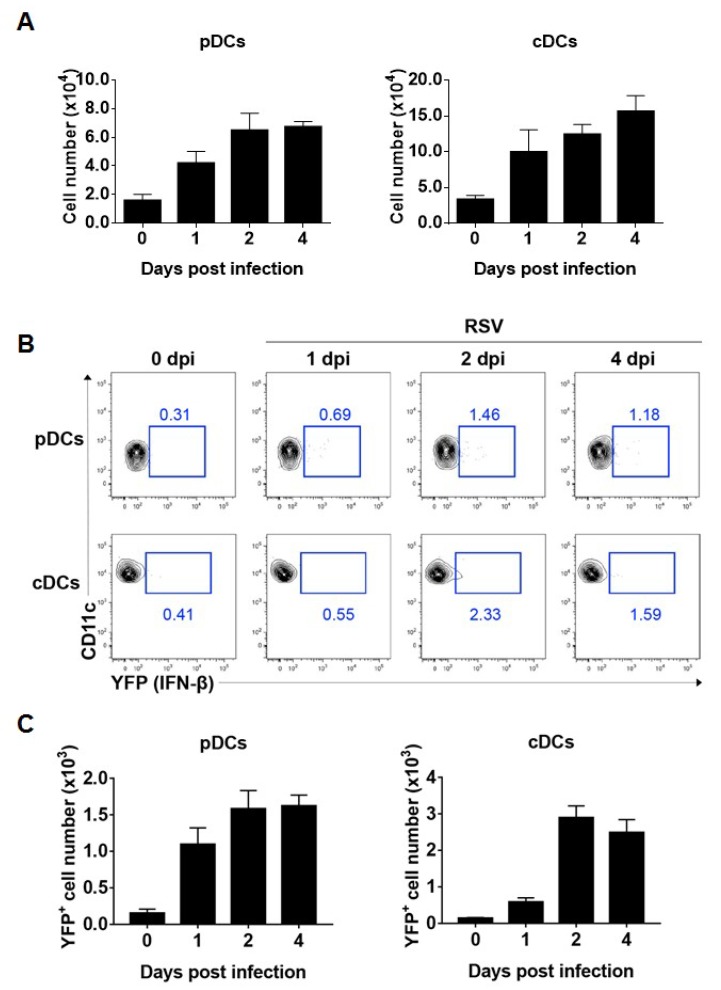
cDCs and pDCs in the lung can produce interferon-β during RSV infection. (**A**–**C**) Interferon-β/YFP reporter (WT) mice were infected intranasally with 1.0 × 10^7^ pfu RSV. After the indicated number of days post-infection, lung cells were isolated from mice and analyzed for interferon-β expression by flow cytometry. (**A**) The absolute number of lung pDCs and cDCs per lung was calculated. (**B**,**C**) Representative results are displayed as the number of YFP^+^ cells per lung. The results are representative of three independent experiments.

Both pDCs and DCs reside in lung mucosa and sense the presence of foreign antigens [21,22]. Both cell types are required for type I interferon production during RSV mucosal infection [8]. To determine whether pDCs or DCs produce interferon-β in the respiratory mucosa during RSV infection in vivo, we intranasally inoculated interferon-β/YFP reporter mice with RSV and analyzed RSV-infected innate immune cells in the lung. As a result, lung infiltrated pDCs and cDCs were increased after RSV infection (Figure 2A), and both pDCs and DCs produced interferon-β between 1 and 4 days post-infection (Figure 2B). After lung RSV infection, DCs capture RSV and moved into draining lymph nodes for T cell activation, but pDCs accumulate in the RSV-infected lung and secrete type I IFN [23]. Because such characteristics developed and considering both frequency and cell number (Figure 2B,C), we conclude that cDCs and pDCs are sufficient producers of interferon-β in the lung during RSV infection in vivo.

### 3.3. RSV Induces pDCs to Produce Interferon-β via the TLR7-MyD88- and IFNα1R-Dependent Pathway 

**Figure 3:**
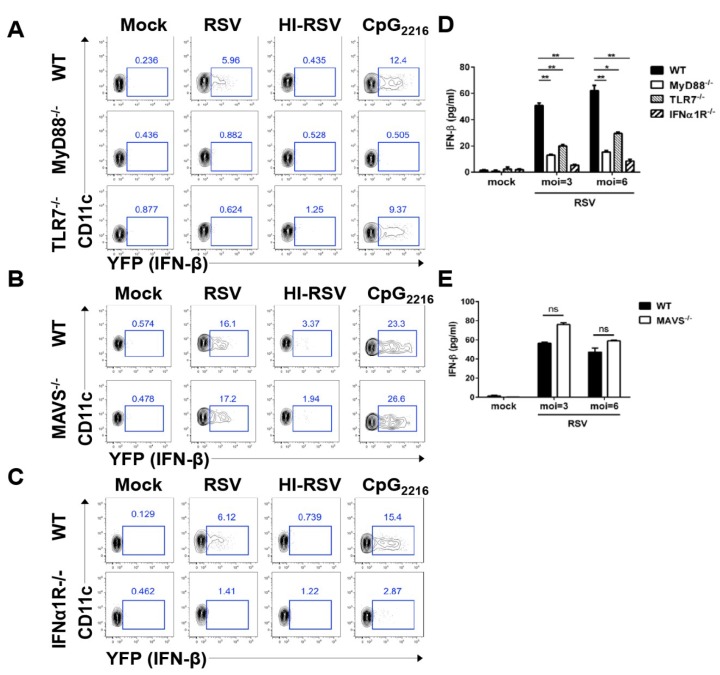
Interferon-β production is dependent on the MyD88-mediated pathway in pDCs in vitro. (**A**–**C**) BM cells from interferon-β YFP/reporter (WT), MyD88-deficient interferon-β/YFP reporter (MyD88^−/−^), and TLR7-deficient interferon-β/YFP reporter (TLR7^−/−^) mice (**A**), MAVS-deficient interferon-β/YFP reporter (MAVS^−/−^) mice (**B**), or interferon-α1R-deficient interferon-β/YFP reporter (interferon-α1R−/−) mice (**C**) were infected with RSV at a multiplicity of infection (MOI) of 3, heat-inactivated RSV (HI-RSV) at an MOI of 3, or with 2.5 μg/mL CpG_2216_. After an 18-h stimulation, cells were harvested and analyzed for interferon-β expression by flow cytometry. (**D**,**E**) The supernatant was collected from the BM cell culture 18 h after stimulation and analyzed for interferon-β by ELISA. The results are representative of three independent experiments. Data are presented as the mean ± SEM. * *P* < 0.05, ** *P* < 0.01, and *** *P* < 0.001 as calculated by the Student’s *t* test.

To determine which signaling pathway is activated by RSV to drive type I interferon production, we crossed interferon-β/YFP reporter mice with MyD88-, TLR7-, IFNα1R-, or MAVS-deficient mice and tracked interferon-β-producing BM cells. pDCs mainly express TLR7 and TLR9 in the endosomal compartment [24]. In response to RSV infection, neither TLR7- nor MyD88-deficient pDCs produced interferon-β, whereas MAVS-deficient pDCs did (Figure 3A,B). In addition, interferon-α1R-deficient pDCs did not actively produce interferon-β upon RSV stimulation (Figure 3C). Next, we measured interferon-β levels in the supernatants of RSV-infected BM cells from MyD88-, TLR7-, IFNα1R-, and MAVS-deficient mice to determine whether these BM cells actually secreted interferon-β in vitro. In response to RSV infection, MyD88- and TLR7-deficient BM cells exhibited impaired interferon-β production (Figure 3D), as did IFNα1R-deficient BM cells, suggesting that interferon-β induction is dependent upon the TLR7-MyD88 pathway and interferon-αR signal in BM cells. However, interferon-β production was unaffected by MAVS-deficient BM cells during RSV infection in vitro (Figure 3E).

### 3.4. pDCs Contribute to the Cytotoxic T Cell Response

**Figure 4:**
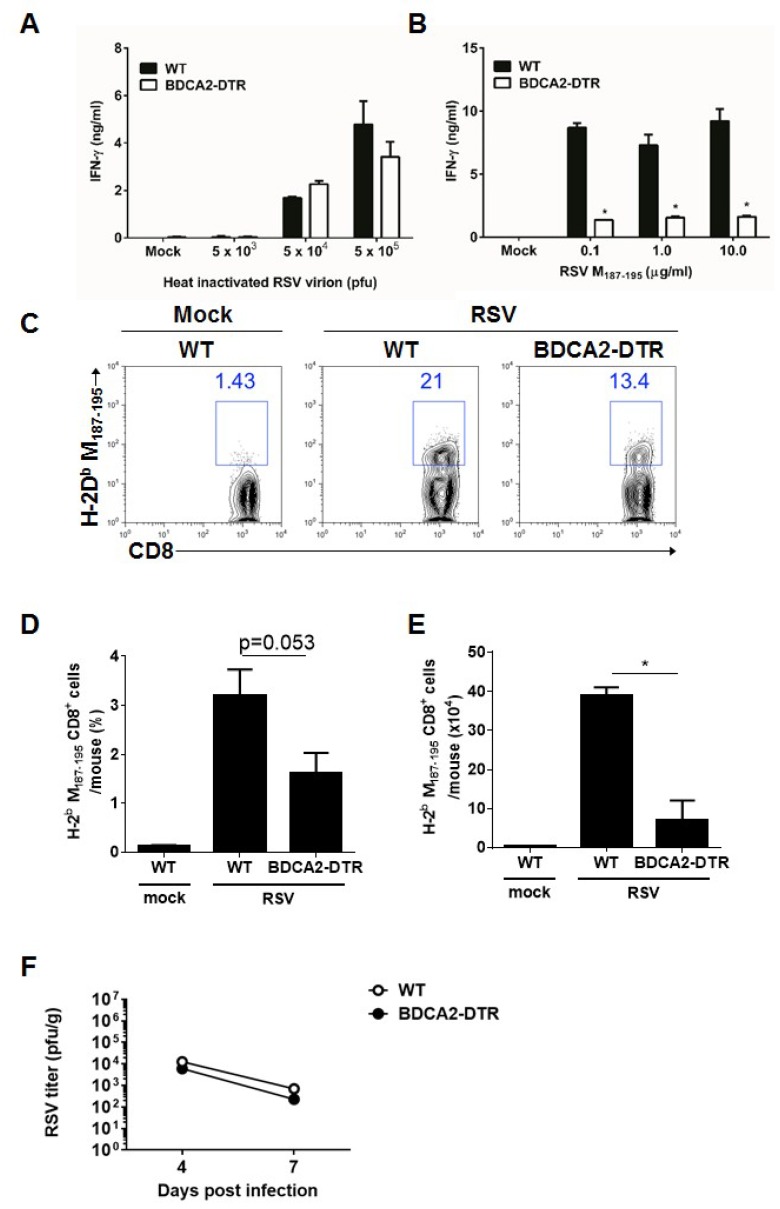
Plasmacytoid dendritic cells are needed for proper cytotoxic T cell responses to RSV infection, while pDC depletion does not alter Th1 induction and the virus titers in RSV-infected mice. (**A**,**B**) Diphtheria toxin-treated wild-type and BDCA2-DTR mice were infected intranasally with 1.0 × 10^7^ pfu RSV. At 8 days post-infection, CD4^+^ and CD8^+^ T cells were isolated from spleens and stimulated with irradiated APCs with the indicated amount of heat-inactivated virion or RSV M_187–195_ peptide for 72 h, respectively. Interferon-γ production from CD4^+^ T cells (**A**) and CD8^+^ T cells (**B**) was measured by ELISA. (**C**–**E**). The indicated groups of mice were infected with 1.0 × 10^7^ pfu RSV, and RSV M_187–195_ peptide-specific CD8^+^ T cells were detected by flow cytometry in the lung 8 days post-infection (**C**). Frequency (**D**) and cell number (**E**) of RSV M187–195 peptide-specific CD8^+^ T cells are shown on the bar graphs. (**F**) WT and BDCA2-DTR mice (*n* = 5 per group) were intraperitoneally administered with 250 ng/mouse of DT at day −1, +1, +3 and intranasally infected with 1.0 × 10^7^ pfu of RSV at day 0. After the indicated days post-infection, lung homogenates were harvested and RSV titers were determined by plaque assay. The data are presented as the mean ± SEM. The results are representative of two independent experiments.

Type I IFNs is known to important cytokines for CD8^+^ T cell activation [25]. Also, pDCs play an critical role in CD8^+^ T cell activation through type I IFN secretion [26]. To investigate the role of pDCs in the adaptive T cell response against RSV infection, we used BDCA2-DTR mice for depletion of pDCs. After the BDCA2-DTR mice were depleted of their pDCs by treatment with diphtheria toxin, the mice were infected with RSV. pDC-depleted mice did not show any impairments in their CD4^+^ T cell responses but exhibited a considerably reduced cytotoxic T cell response (Figure 4A,B). Further, RSV-infected lungs from pDC-deleted mice had a decreased frequency and cell number of RSV M_187–195_ peptide-specific CD8^+^ T cells (Figure 4C–E). Next, we compared RSV titers during RSV infection at day 4 and 7 post infection. pDC-depleted BDCA2-DTR mice showed comparable RSV titers in lung (Figure 4F), and these results indicated that Th1 responses in pDC-depleted mice were sufficient to clear viruses while CTL responses were considerably reduced in this mice. Thus, these findings suggest that pDCs play a crucial role in the induction of the adaptive CTL priming response, as well as type I interferon production in early innate immunity. 

## 4. Discussion

In this study, we examined major interferon-β responses to mucosal RSV infection by tracking interferon-β-producing cells. Our results demonstrate that pDCs are rapidly recruited to sites of inflammation and become the major interferon-β-producing cell population in RSV-infected lungs. These results are consistent with the finding that pDCs are the major interferon-β-producing cell population in RSV-stimulated BM cells in vitro. During RSV infection, pDCs require the MyD88 pathway, but not the MAVS pathway, to produce interferon-β. Furthermore, pDCs must also induce cytotoxic CD8^+^ T cell responses during RSV infection. 

Type I interferon is generally considered a key cytokine for the regulation of the antiviral response in innate immunity [27]. Earlier reports indicated that pDCs produce type I interferon during viral infection [28,29], and pDC depletion aborted IFN-α following RSV infection in PBMC [30]. Among the immune cell populations of BM cells, pDCs and DCs have the capacity to produce interferon-β in response to RSV infection. Also, our in vivo results suggest that pDCs are major interferon-β producers during RSV infection. Since dendritic cell quickly migrate in to draining lymph node after RSV infection in vivo, it is presumed that lung reside pDCs were mainly involved in type I IFNs production in vivo. Also, recent studies showed that alveolar macrophages produce type I interferon during early periods of infection [17,31]. It is thus necessary to study the role of alveolar macrophages in early innate immunity during RSV infection.

Moreover, our findings suggest that pDCs contribute to both the CD8^+^ T cell immune response and innate immunity during RSV infection. A recent study using BDCA2-DTR mice, a conditional knockout model used for the depletion of pDCs, indicated that pDCs influence viral-specific T cell responses during systemic MCMV or VSV infection [15]. In addition, another report showed that pDCs are important for the CD8 T cell response as well as early type I interferon production during systemic, but not local, HSV infection [32]. The detailed role of pDCs in antiviral immunity during RSV infection requires further investigation.

Each immune cell type expresses different PRRs [33,34] that can contribute to cell type-specific antiviral responses to RSV [35]. Our in vitro BM results demonstrate that pDCs produce interferon-β via TLR7-MyD88-mediated signaling, possibly due to the predominant expression of TLR7 in pDCs. These results were consistent with previous reports that accentuated rodent-specific pneumovirus induced type I interferon responses require TLR7/MyD88 pathway [36]. A previous study demonstrated that MAVS is essential for innate immunity, but not for cytotoxic T cell responses, to RSV infection [37]. However, a recent study showed that IPS-1 signaling plays a nonredundant role in mediating antiviral responses and viral clearance [38]. The dissimilarities of our results from those of previous studies might be attributable to different expression levels of basal and inducible PRRs in each cell population.

The type I interferon feedback mechanism plays an important role in amplifying early antiviral responses. Akira et al. [39] showed that this feedback pathway represses NDV viral replication in pDCs. Meanwhile, our findings indicated that RSV does not induce interferon-β production in interferon receptor-deficient pDCs in BM. Although a deficiency in type I interferon feedback induces viral replication, the RIG-I-MAVS system does not play a key role in viral recognition or production of type I interferon because RSV is primarily recognized via TLR-MyD88, and not MAVS, in pDCs. However, the mechanism of interaction by which type I interferon and TLR-MyD88- or MAVS-mediated signaling play roles in RSV infection remains unclear.

Adaptive T cell responses are essential for effective viral clearance [40]. Type I interferon modulates the level of gene expression involved in innate immunity and initiates the adaptive immune response [41]. Further, type I interferon stimulates a general antiviral environment by activating NK cells and CD8^+^ T cells [15]. Previous results showed that pDC depletion enhances Th2 immune responses, suggesting that pDCs may be involved in adaptive immunity [19]. Also, TLR7 pathway in pDCs were associated with host protection against rodent-specific pneumovirus infection [36]. We also confirmed the role of pDCs in adaptive immune responses during RSV infection using BDCA2-DTR mice. Our results suggest that pDCs are required for proper CD8^+^ T cell responses in vivo and play an important role in adaptive as well as innate immune responses to RSV infection

In conclusion, our study demonstrates the requirement for pDCs to produce MyD88-dependent interferon-β during the development of adaptive immune responses to RSV infection. These data have significant implications in the design of vaccines and management of RSV infection. Agents that stimulate pDCs may be ideal adjuvants for RSV vaccines to confer protective immune responses.
